# SignaLink3: a multi-layered resource to uncover tissue-specific signaling networks

**DOI:** 10.1093/nar/gkab909

**Published:** 2021-10-11

**Authors:** Luca Csabai, Dávid Fazekas, Tamás Kadlecsik, Máté Szalay-Bekő, Balázs Bohár, Matthew Madgwick, Dezső Módos, Márton Ölbei, Lejla Gul, Padhmanand Sudhakar, János Kubisch, Oyebode James Oyeyemi, Orsolya Liska, Eszter Ari, Bernadette Hotzi, Viktor A Billes, Eszter Molnár, László Földvári-Nagy, Kitti Csályi, Amanda Demeter, Nóra Pápai, Mihály Koltai, Máté Varga, Katalin Lenti, Illés J Farkas, Dénes Türei, Péter Csermely, Tibor Vellai, Tamás Korcsmáros

**Affiliations:** Earlham Institute, Norwich NR4 7UZ, UK; Department of Genetics, ELTE Eötvös Loránd University, Budapest H-1117, Hungary; Earlham Institute, Norwich NR4 7UZ, UK; Department of Genetics, ELTE Eötvös Loránd University, Budapest H-1117, Hungary; Department of Genetics, ELTE Eötvös Loránd University, Budapest H-1117, Hungary; Earlham Institute, Norwich NR4 7UZ, UK; Earlham Institute, Norwich NR4 7UZ, UK; Department of Genetics, ELTE Eötvös Loránd University, Budapest H-1117, Hungary; Earlham Institute, Norwich NR4 7UZ, UK; Gut Microbes and Health Programme, Quadram Institute Bioscience, Norwich, NR4 7UQ, UK; Earlham Institute, Norwich NR4 7UZ, UK; Gut Microbes and Health Programme, Quadram Institute Bioscience, Norwich, NR4 7UQ, UK; Earlham Institute, Norwich NR4 7UZ, UK; Gut Microbes and Health Programme, Quadram Institute Bioscience, Norwich, NR4 7UQ, UK; Earlham Institute, Norwich NR4 7UZ, UK; Earlham Institute, Norwich NR4 7UZ, UK; Translational Research in GastroIntestinal Disorders, Leuven BE-3000, Belgium; Department of Genetics, ELTE Eötvös Loránd University, Budapest H-1117, Hungary; Zeekay Institute of Advanced and Professional Studies, Lagos, Nigeria; Department of Genetics, ELTE Eötvös Loránd University, Budapest H-1117, Hungary; HCEMM-BRC Metabolic Systems Biology Lab, Szeged H-6726, Hungary; Synthetic and Systems Biology Unit, Institute of Biochemistry, Biological Research Centre, Eötvös Loránd Research Network (ELKH), Szeged H-6726, Hungary; Doctoral School in Biology, University of Szeged, Szeged H-6720 Hungary; Department of Genetics, ELTE Eötvös Loránd University, Budapest H-1117, Hungary; HCEMM-BRC Metabolic Systems Biology Lab, Szeged H-6726, Hungary; Synthetic and Systems Biology Unit, Institute of Biochemistry, Biological Research Centre, Eötvös Loránd Research Network (ELKH), Szeged H-6726, Hungary; Department of Genetics, ELTE Eötvös Loránd University, Budapest H-1117, Hungary; Department of Genetics, ELTE Eötvös Loránd University, Budapest H-1117, Hungary; ELKH/MTA-ELTE Genetics Research Group, Budapest H-1117, Hungary; Department of Genetics, ELTE Eötvös Loránd University, Budapest H-1117, Hungary; Department of Genetics, ELTE Eötvös Loránd University, Budapest H-1117, Hungary; Department of Morphology and Physiology, Semmelweis University, Budapest H-1088, Hungary; Department of Genetics, ELTE Eötvös Loránd University, Budapest H-1117, Hungary; Earlham Institute, Norwich NR4 7UZ, UK; Department of Genetics, ELTE Eötvös Loránd University, Budapest H-1117, Hungary; Department of Genetics, ELTE Eötvös Loránd University, Budapest H-1117, Hungary; Institute of Molecular Biotechnology, Vienna A-1030, Austria; Centre for the Mathematical Modelling of Infectious Diseases (CMMID), London School of Hygiene & Tropical Medicine, London WC1E 7HT, UK; Department of Genetics, ELTE Eötvös Loránd University, Budapest H-1117, Hungary; Department of Morphology and Physiology, Semmelweis University, Budapest H-1088, Hungary; Citibank Europe plc Hungarian Branch Office, Budapest H-1133, Hungary; Heidelberg University, Faculty of Medicine, and Heidelberg University Hospital, Institute for Computational Biomedicine, Bioquant, Heidelberg, Germany; Department of Molecular Biology, Semmelweis University, Budapest H-1094, Hungary; Department of Genetics, ELTE Eötvös Loránd University, Budapest H-1117, Hungary; ELKH/MTA-ELTE Genetics Research Group, Budapest H-1117, Hungary; Earlham Institute, Norwich NR4 7UZ, UK; Department of Genetics, ELTE Eötvös Loránd University, Budapest H-1117, Hungary; Gut Microbes and Health Programme, Quadram Institute Bioscience, Norwich, NR4 7UQ, UK

## Abstract

Signaling networks represent the molecular mechanisms controlling a cell's response to various internal or external stimuli. Most currently available signaling databases contain only a part of the complex network of intertwining pathways, leaving out key interactions or processes. Hence, we have developed SignaLink3 (http://signalink.org/), a value-added knowledge-base that provides manually curated data on signaling pathways and integrated data from several types of databases (interaction, regulation, localisation, disease, etc.) for humans, and three major animal model organisms. SignaLink3 contains over 400 000 newly added human protein-protein interactions resulting in a total of 700 000 interactions for *Homo sapiens*, making it one of the largest integrated signaling network resources. Next to *H. sapiens*, SignaLink3 is the only current signaling network resource to provide regulatory information for the model species *Caenorhabditis elegans* and *Danio rerio*, and the largest resource for *Drosophila melanogaster*. Compared to previous versions, we have integrated gene expression data as well as subcellular localization of the interactors, therefore uniquely allowing tissue-, or compartment-specific pathway interaction analysis to create more accurate models. Data is freely available for download in widely used formats, including CSV, PSI-MI TAB or SQL.

## INTRODUCTION

Signaling pathways describe the chain of molecular events of a cell in response to various external or internal signals. Regulation of many physiological cell functions requires these signaling pathways to work precisely. Deregulation of pathways can lead to disturbances in cellular functions resulting in various diseases. Therefore, it is important to analyse and understand these regulatory processes in order to uncover the workings of these diseases (1).

Traditionally, signaling pathways were considered as a linear chain of events, rather than a complex network of intertwining interactions. However, from a functional viewpoint, to provide a biologically relevant representation of the cell's inner workings we cannot differentiate individual biological processes as independent pathways, as these processes have many common interactors and cross-talks between them (2). Therefore, in the past decade signaling research started to view biological processes as one single signaling network, which includes and represents multiple pathways (3). Although there have been many advances in this field, most signaling resources still only represent a subset of this complex network.

Currently available signaling databases use interaction data from high-throughput experiments and text mining, while others such as SIGNOR (4) and Reactome (5) use manual curation from literature with differing protocols. Most of these resources create a detailed representation of one specific aspect of the regulatory mechanisms of signaling pathways, therefore often leaving out part of the complex network of signaling events. Different levels of detail also make it difficult to create comparative analyses. Therefore, we have previously developed SignaLink aiming to represent signaling networks as connected functional pathways. With SignaLink1 (6), we created a network resource of major signaling pathways by applying uniform manual curation protocols for two model species and humans, and systematically uncovered pathway cross-talks. In SignaLink2 (7), we introduced the concept of representing signaling pathways and their regulatory interactions in a novel multi-layered (onion-like) structure. This approach extends signaling pathways with their regulators, represented in separate layers. By defining connections between layers, we are able to represent a more complete network of regulatory mechanisms, creating a biologically meaningful model of signaling networks (8). SignaLink has since provided researchers a map of global signaling pathways to better understand and analyze the regulation of signaling pathways and pathway cross-talks. Accordingly, SignaLink has been a widely used tool in signaling research with over 4000 downloads from 400 institutes in 81 countries since 2013.

As advancements in the field emerge, integrating new data has been a priority in the development of SignaLink. Hence, we now present SignaLink3, the newest version of SignaLink. Next to *Homo sapiens*, we provide signaling data on three main model organisms—*Caenorhabditis elegans, Drosophila melanogaster* and *Danio rerio—*in a single web resource. Pathway components are represented at the core of the resource, while their regulatory proteins, transcription factors, miRNAs, and newly added lncRNAs make up each layer of the multi-layered structure. In the update, integrated expression data provides researchers a way to analyze tissue specificity and subcellular localization of signaling networks. SignaLink's new website was developed based on feedback from users to provide an easy-to-use platform with new features and various download formats.

## DESCRIPTION AND CONTENT

**Figure 1.**
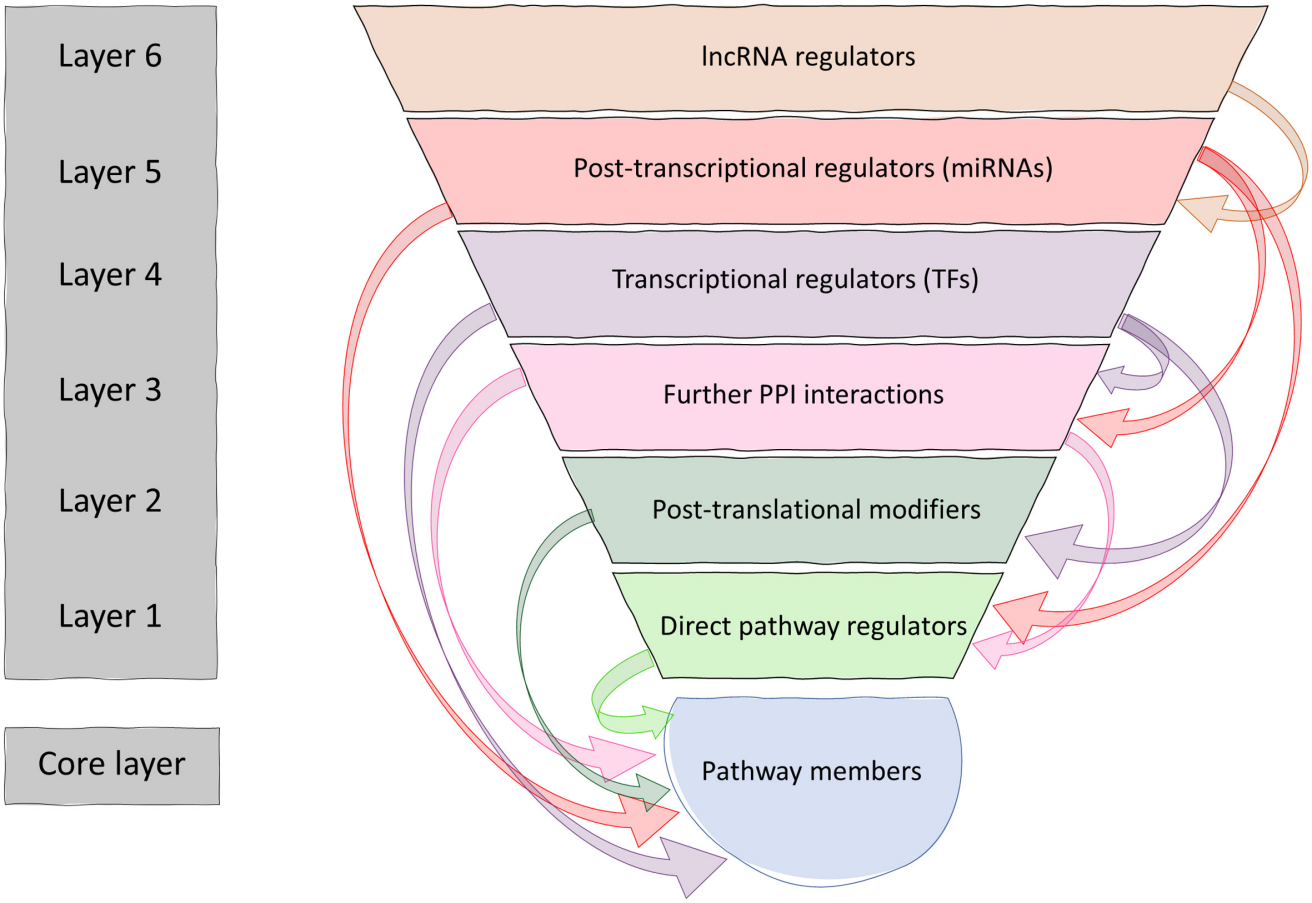
The multilayered structure of SignaLink3. Pathway members and their interactions make up the core of SignaLink. These pathways are extended by their: direct regulators (proteins that are often not part of the canonical pathway membership), post-translational modifiers (enzymes that could modify signaling proteins), protein-protein interactions (PPIs), transcriptional and post-transcriptional regulators (transcription factors, miRNAs, and lncRNAs) This multilayered arrangement represents the complex interactions that regulate the signaling pathways.

**Figure 2.**
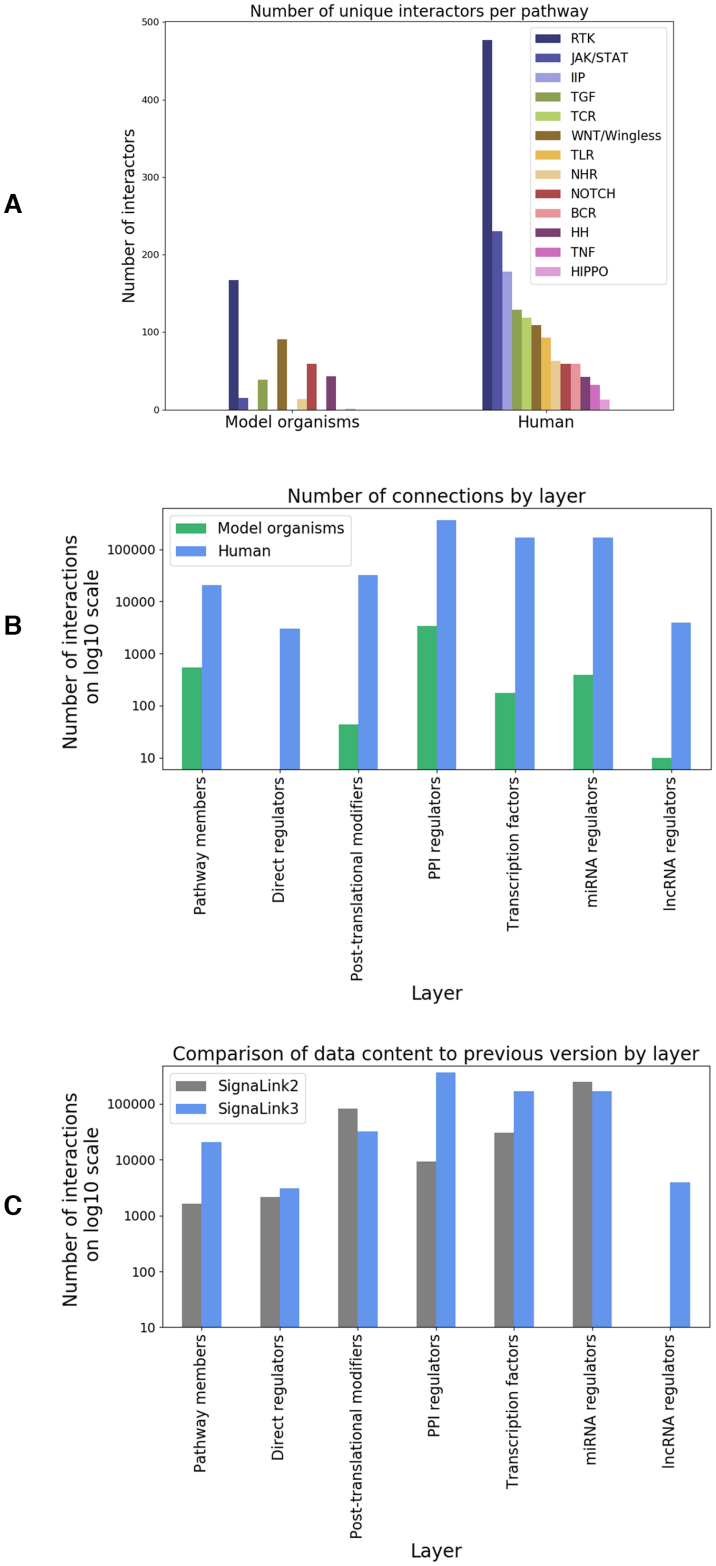
Statistics of SignaLink3 data contents. (**A**) Number of unique interactors (proteins) involved in each pathway included in SignaLink3 in humans and three model organisms combined (non-standard abbreviations - IIP: innate immune pathway, TCR: T-cell receptor pathway, BCR: B-cell receptor pathway, HH: Hedgehog pathway). (**B**) Number of unique connections (interactions) in each layer in humans and three model organisms combined. (**C**) Number of human interactions in each layer of SignaLink3 compared to the previous version. The decrease in the number of interactions in SignaLink3 in the PTM and miRNA layers are due to the fact that SignaLink3 does not contain any predicted interactions, only experimentally verified ones.

With the previous version of SignaLink, we have introduced the novel concept of a multi-layered signaling network. We still adhere to this concept in our update due to its efficiency of representing biological processes. The aim of this approach is to represent regulatory mechanisms of signaling pathways as a whole network, rather than separate entities as most other resources do. In the multi-layered structure (Figure 1), the core represents the signaling proteins involved in the 13 pathways included in SignaLink3 and connections between these pathway proteins compiled together using manual curation. For the previous versions, this curation was done in-house. For SignaLink3, we not only updated our own curation but also integrated external curation data from a few selected pathway resources (such as SIGNOR (4) and Reactome (9)) that applied standardized curation protocols very similar to the one we have been using (8). The curated pathways in the core of SignaLink3 contain the original major signaling pathways: RTK (Receptor Tyrosine Kinase, containing all MAPK and Insulin subpathways), TGF-β, WNT, Hedgehog, JAK/STAT, Notch and NHR (Nuclear Hormone Receptor) pathway as well as five newly added key pathways: B- and T-cell receptor, Hippo, Toll-like receptor, and innate immune pathways (Figure 2). Starting with SignaLink1, the pathway definitions follow the evolution-based concept of Pires-daSilva and Sommer (10), and now are more complete than in the previous versions. Pathway membership annotation within SignaLink allows for the identification of multi-pathway proteins to uncover pathway cross-talks that are otherwise not clear. As revealed by SignaLink's previous version these cross-talks are important in modeling signaling networks in drug-target discovery and uncovering their pathological role in diseases (11).

After establishing the core, signaling pathways are extended by using protein-protein and regulatory interaction resources to create the rest of the onion-like structure. Direct pathway regulators such as scaffold and endocytic proteins obtained from literature curation form the first layer. Proteins in Layer 1 are key for the spatial regulation of the signaling flow but often left out from traditional pathway maps (12). Post-translational modifying enzymes from PTM-specific resources (e.g. PhosphoSite (13)) make up the second layer (Figure 1, Layer 2). As compared to the previous version (7), SignaLink3 only contains experimentally verified interactions. This results in a more reliable and accurate network resource. Therefore, the number of connections in the post-translational layer decreased, as SignaLink2 also contained predicted interactions (Figure 2C). Proteins in Layer 2 are mostly responsible for the temporal regulation of the signaling flow. A major update to SignaLink3 is the large number of directed protein-protein interactions (PPIs) that were integrated from large-scale databases such as OmniPath (14), BioGRID (15), ComPPI (16,17) and IntAct (17) to the third layer (Figure 1, Layer 3). In Layer 3, SignaLink3 now contains more than 350,000 PPIs for pathway proteins (in humans, Figure 2) as compared to the ∼3000 included in the previous version (Figure 2C). All manually curated interactions in SignaLink3 are directed (including the interactions in Layer 1 and Layer 2), additionally we also provide the direction of the PPIs in Layer 3 where possible. This is fundamental, as direction of interactions are crucial in signal flow (18). Hence, we have included predictions for both the direction (which protein has an effect on which) and signage (*e.g*., stimulatory or inhibitory) of the interactions. As a result, of all interactions of SignaLink3 94.3% are directed and 24.2% are signed. In the case of direction prediction, we used methods developed by Liu *et al.* and Rhodes *et al.* (19,20). We set up a training set based on the directed interaction data from the Reactome database, and created a scoring system calculated from the directions of domain-domain interactions of the proteins. By applying these scores to the PPIs, we predicted the direction of more than 300,000 undirected interactions over the four species. With these predictions we could provide a more accurate and detailed signaling network (Supplementary Figure S1). The sign of an interaction, i.e. the effect each protein has on its target (stimulation or inhibition) is also an important aspect of interactions in signaling networks. Therefore, we applied the method of Vinayagam *et al.* (21) based on RNAi screens to predict the signage of the protein-protein interactions where such information was not previously supplied in the source databases. For each interacting pair we calculated the total number of positive and negative correlations and assigned a score determining whether the interaction means a positive or a negative regulation. With this method we predicted the effect of 116 interactions. Information on the type of interaction (i.e. phosphorylation, co-localization), interaction detection methods and other annotations were also imported from the integrated resources (e.g. InnateDB (22), TFlink (https://tflink.net/), TarBase (23)) where available.

The next three layers (Figure 1, Layers 4–6), contain transcriptional and post-transcriptional regulators. The fourth layer contains transcription factors known to regulate genes encoding proteins in the inner layers (Core and Layer 1–3). The number of transcriptional regulatory interactions increased in SignaLink3 by more than 130,000 connections (Figure 2C), resulting in altogether 168 817 regulatory connections for the four species (using data from TFlink (https://tflink.net/)). Two major novelties of SignaLink3 is that we doubled the number of miRNAs (Figure 1, Layer 5) integrated from miRDeathDB (24), miRecords (25), miR2Disease (26), TarBase (23) and starBase (27), and we included experimentally verified lncRNAs as well from databases such as lncRInter (28), NPInter (29) and starBase (27). Regulation by lncRNAs were not included in previous versions of our resource (Figure 2C). These lncRNAs in Layer 6 mediate signaling pathways both transcriptionally and post-transcriptionally. By associating lncRNAs to pathways and diseases, SignaLink3 provides a comprehensive tool for current signaling research studies.

SignaLink3 integrates signaling data on three major model organisms next to humans. While compared to humans, less data is available for model organisms - especially for lncRNAs, we aimed to include as much data for the other species as possible (Figure 2B). SignaLink3 is the largest signaling resource for *D. melanogaster* with over 1400 unique proteins and 30 miRNAs. Next to *D. melanogaster* SignaLink is the only currently available signaling network resource for *C. elegans* as well as *D. rerio* with the full integration of our previously separate resource, SignaFish (30). Annotations of pathways are also sparser in model organisms than in humans, however pathways such as RTK, WNT/Wingless and Notch can be explored in detail in these species as well (Figure 2A). By providing data in a unified format on multiple species in a single resource, we make signaling research more accessible for scientists working on model species and comparative, evolutionary studies (31).

Recent advancements in transcriptomics have revealed that many functionally and pathologically important molecular mechanisms can differ based on the expression of a gene in a given tissue or the localization of the protein within the cell. Therefore, creating tissue-specific signaling networks could pave the way in modeling and drug discovery for tissue-specific diseases and cancers. Hence, a major advantage of SignaLink3 is the recently integrated gene expression data with our signaling and regulatory networks. This provides the novel approach of analyzing signaling networks in a tissue-specific manner. We retrieved tissue-specific gene expression data for all four species in SignaLink from the Bgee database (31) and combined it with our network data. As a result, our users can directly access and filter information about the tissue specificity of each of the proteins present in SignaLink3. By providing this data, researchers can reconstruct tissue-specific signaling networks, discover unique regulatory mechanisms, and model pathologically significant differences in specific tissues. SignaLink3 also contains major and minor cellular localization information for each interaction using data from the ComPPI database (17), which we developed earlier. Using this data, users can filter biologically unlikely PPIs and create models of compartment-specific biological functions.

## CASE STUDY

**Figure 3.**
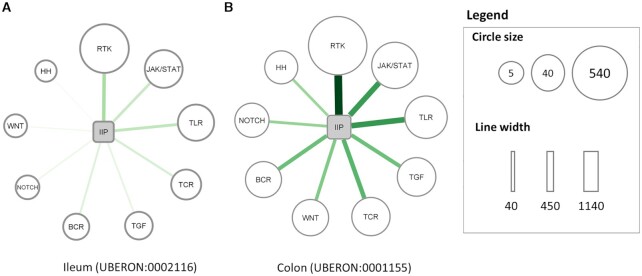
Number of cross-talks of the innate immune pathway. (**A**) Number of cross-talks between IIP and other pathways in SignaLink3 in the ileum. (**B**) Number of cross-talks between IIP and other pathways in SignaLink3 in the colon. Width and darkness of lines indicate the number of cross-talks between the connected pathways. The wider and darker the line is, the more cross-talks there are. The size of the circles indicates the number of unique proteins involved in the cross-talk with IIP. The size of the IIP node is fixed and does not hold any information (non-standard abbreviations: IIP: innate immune pathway, TCR: T-cell receptor pathway, BCR: B-cell receptor pathway, HH: Hedgehog pathway).

**Figure 4.**
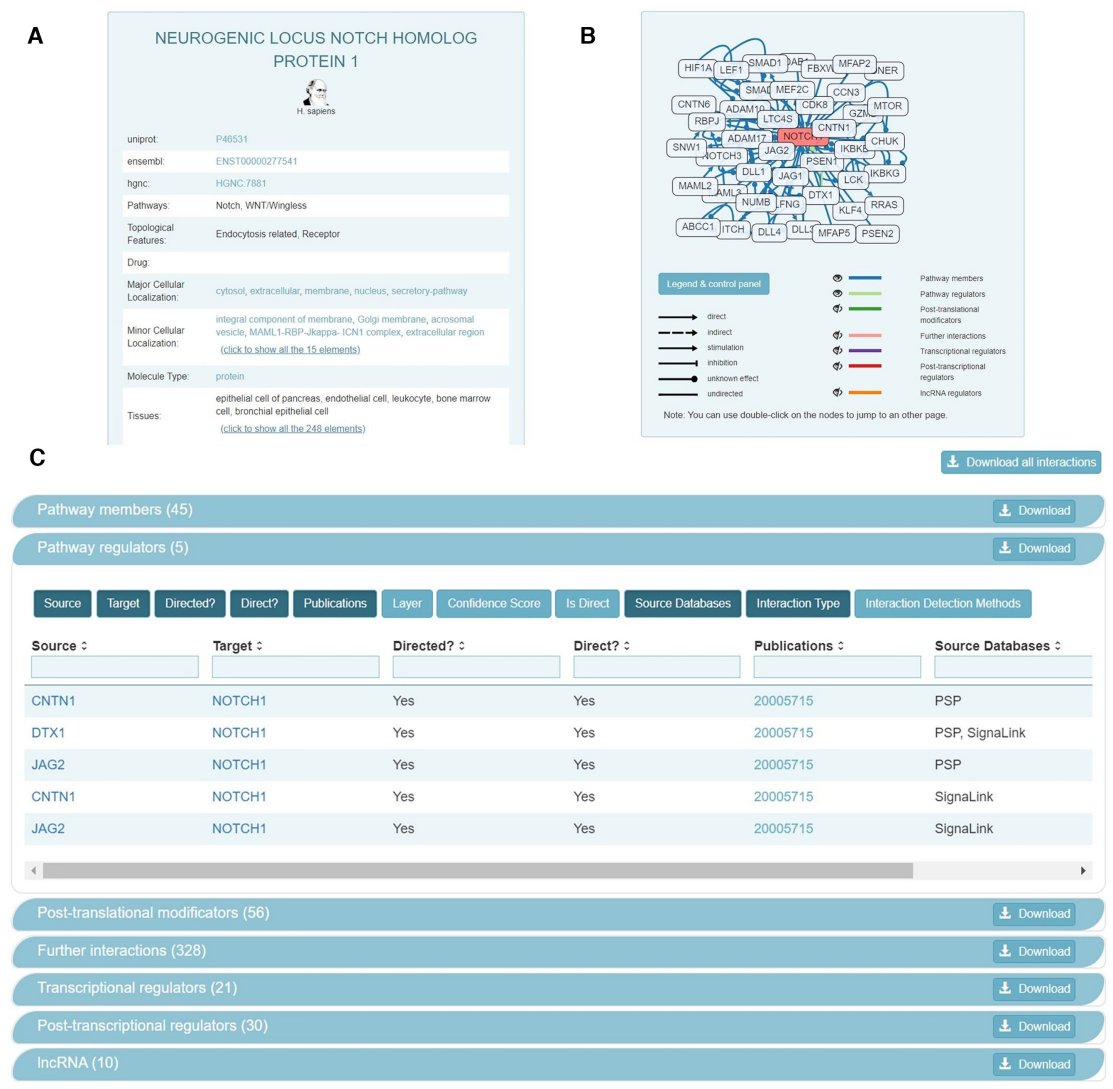
Updated protein page design of SignaLink3. (**A**) Basic information on a protein with multiple identifiers and cross-links. Annotated by the pathway, topological features, molecule type and subcellular localization as well as tissue expression. (**B**) Interactive network representation of the connections of the protein. Users can choose which type of interactions they would like to visualize. (**C**) Further information on the connections of the protein. As a novel feature, connections can be individually downloaded by category or all connections of the protein can be obtained by clicking the ‘Download all interactions’ button.

To demonstrate the new, tissue-specific analysis feature of SignaLink3, we compared the level of cross-talks between pathways in different tissue types (Figure 4). By using the customizable selection options on the download page, we selected interactions between proteins involved only in the newly added innate immune pathway (IIP) of *H. sapiens*. With the usage of the new tissue filtering function, we only downloaded interactions present in either the ileum or the colon. From the exported data file, we calculated the number of cross-talks between the innate immune pathway and other pathways included in SignaLink3. We followed the cross-talk definition from previous versions of SignaLink (7): if one protein of the interaction was annotated to be involved in the pathway of interest (innate immune pathway in this case), while its interacting pair was not, we considered this as a cross-talk between pathways. By carrying out the same calculations on different tissues, we set up a connection map of the number of cross-talks of the innate immune pathway (Figure 3).

As seen from the figure, the number of cross-talks differs between tissues. We had a significantly larger number of cross-talks overall in colonic tissues, however it is important to note that in the retrieved dataset a much higher percentage of interactions were annotated to be expressed in the colon as in the ileum. However, we also saw slight differences in the relative number of cross-talks in the two tissues. For example cross-talks of the innate immune pathway with the WNT pathway was higher ranked in the colon, while lower ranked in the ileum (Figure 3). Such analysis can reveal unique signaling networks of distinct tissues, and can be used in understanding disease related alterations. Differences between the signaling networks in the ileum and colon could be used in uncovering tissue specific aspects of gut diseases such as differences in the two types of inflammatory bowel disease: ulcerative colitis and Crohn's disease.

We can conclude that SignaLink3 is a useful resource for creating tissue-specific signaling networks as well as discovering pathway cross-talks, providing a tool for comparing tissues and potentially uncovering the workings of tissue specific diseases. This demonstrative analysis also shows the use and importance of new features of SignaLink3. By using the customizable download page, users can avoid additional data cleaning and filtering steps in their pipeline, which helps in speeding up the analysis.

## DATABASE STRUCTURE AND WEBSITE

Alongside newly added features with regards to data content, SignaLink3 also presents a cleaner, more user-friendly web interface compared to its previous versions. Since the last update, based on feedback and suggestions from users of SignaLink, we aimed to meet our target audience's needs in order to provide an easy-to-use tool for both computational and experimental researchers.

SignaLink3 data is stored in a unified SQLite format. Each of the interactors are classified by molecule type (proteins, miRNAs, lncRNAs) and annotated by the pathway they are involved in, as well as their topological features (e.g. ligand, receptor, transcription factor). Each protein is further annotated with their major and minor cellular localization (16), and tissue localization based on integrated expression data (Figure 4A). Tissues are also represented by their UBERON identifiers (32) to provide an unambiguous representation. A unified identifier system is used in the case of proteins, miRNAs and lncRNAs as well. Proteins are represented by their unique UniProt (33) (Swiss-Prot) IDs, although Ensembl (34), HGNC (35) and other alternative accessions from source databases are also provided. miRNAs and lncRNAs are represented by their RNACentral (36) and miRBase (37) identifiers. In the case of model organisms, FlyBase (38), WormBase (39) and Zfin (40) cross-links are available.

Users can access data by navigating to the desired protein's page on the website. The protein page displays an interactive network figure of the interactions of the protein of interest (Figure 4B). Here, the direction and effect are indicated by arrows. Users can customize the view by choosing which layers’ connections they would like to visualize by clicking the eye symbols in the network control panel. Interactions of the protein can be listed and downloaded individually - and tailored to the user's needs - directly from the protein page as a new feature (Figure 4C). Interactions are annotated with the source database the data came from or PubMed IDs of experimental results next to previously mentioned direction and signage among others. Currently the search function is exclusively available to individual proteins, as pathways or tissues cannot be searched for and browsed. Each protein is annotated by which pathway it belongs to or which tissue it is expressed in on the panel shown in Figure 4A. Pathway and tissue filtering is however available at download.

The multi-layered structure of SignaLink not only provides a biologically relevant representation of signaling networks but also caters for customizable download options. On the new download page, users can select any subset of SignaLink, choosing specific species, pathways or layers. With the integrated tissue and subcellular localization data, users can select specific tissues and create a tissue specific network. We also aim to provide a more straightforward way of obtaining data with step-by-step options for customizing the downloaded dataset. SignaLink3 can be downloaded in many widely used formats such as CSV, PSI-MITAB (41) or BioPAX (42). The entire dataset of SignaLink3 can also be downloaded as a complete SQL file.

## DISCUSSION AND FUTURE PLANS

SignaLink3 provides an extended and updated resource of signaling networks for humans and three model species. SignaLink3 builds on the previously developed multi-layered network structure and complements it with novel features and annotations to allow creating cell-compartment or tissue-specific networks. SignaLink3 extends signaling pathways by integrating regulatory connections scattered across different databases in one single resource. The derived multi-layered structure presents a more detailed network of signaling events. Since 2013, the community of SignaLink users has grown, reaching researchers in 81 countries with 25,000 visits. Based on the citations on the publication of the previous version of SignaLink (7), the resource proved to be a useful tool in cancer research (43), drug discovery (44), and signaling pathway research (45). These works emphasize that SignaLink provides useful and easy-to-use features filling the current gaps in signaling network research. Other large-scale data sources such as OmniPath (14) and SIGNOR (Licata *et al.* 2020) integrating SignaLink data also indicate the need for such a comprehensive resource. By cross-linking data with such resources, SignaLink's data can reach a wider platform of users. With the rapidly growing amount of data available, one of our priorities is providing an up-to-date resource for our community. Feedback from users - pointing out missing features or personal preferences - has also been considered in the development of SignaLink3. Accordingly, we provide new, previously missing features for an easier and more complete reconstruction of context-dependent signaling networks.

SignaLink3 is a useful tool for biological modeling, aiding drug discovery research. For these purposes it is crucial to have a thorough annotation of interactions. In SignaLink3, we include predictions for both the direction and effect of interactions, as well as providing information on the type of the interaction (*e.g*., phosphorylation, co-localization, physical association). This additional information allows simulating biochemical reactions and aid in analysis of signal transduction pathways. Another novel feature of SignaLink3 is the possibility of creating tissue specific networks. As pointed out in a review (18) on multilayered signaling network resources - including SignaLink2 - none of the reviewed databases provided tissue specific data on signaling networks (Supplementary Table 1). Since then, a ‘Tissue distribution’ tool has been developed as a part of the Reactome analysis toolset, which makes it possible to create tissue specific signaling networks (5). However, this feature is only available for human signaling proteins while in SignaLink3 we additionally provide tissue annotations for three model species as well. With the integration of gene expression data in the novel version, we were able to further fill the gap of analyzing tissue-specific signaling networks, allowing users to select specific tissues of interest.

With the integrated tissue and cellular localization information, and a large number of newly added interactions, SignaLink3 extends signaling pathways with transcriptional, post-transcriptional, post-translational and now lncRNA regulators in four species, making it one of the largest integrated signaling network resources currently available (Figure 2). With a new, easy-to-use interactive website and customizable download options developed based on user feedback, SignaLink3 is a leading tool in biological modeling, cancer research and understanding diseases such as inflammatory bowel disease (IBD), paving the way in tissue specific network reconstruction and analysis.

In further developments, we aim to include emerging analysis methods and computational approaches in future versions of SignaLink. Our automated pipeline makes it simple to integrate novel data to always provide an up-to-date source of signaling network data with bi-annual data updates. With our growing community, we plan to prioritize user feedback to make SignaLink fit the needs of researchers. As the prevalence of epigenetics research rises, we also plan to integrate epigenetics information, such as methylation profiles or chromatin regulation data to future updates of SignaLink. Currently, transcription factor - target gene interactions mostly consider transcription factor binding sites in the promoter regions of the target genes. In future updates we plan to further extend these binding sites to more distant enhancer regions as well. With the introduction of the option of creating tissue-specific networks we made a leading step in signaling network research. However, with the emergence of single-cell sequencing technologies we plan to provide a way to create cell-type specific networks with further developments.

## DATA AVAILABILITY

SignaLink data is freely available for download in widely used formats, including CSV, PSI-MI TAB or SQL at http://SignaLink.org.

## Supplementary Material

gkab909_Supplemental_File
