# Supplementary material for: SignaLink3: a multi-layered resource to uncover tissue-specific signaling networks
Source: Nucleic Acids Res. 2021 Oct 11;50(D1):D701–9. doi: 10.1093/nar/gkab909 (PMC8728204; doi:10.1093/nar/gkab909)
Supplement: gkab909_Supplemental_File [file gkab909_supplemental_file.pdf]

Supplementary Materials

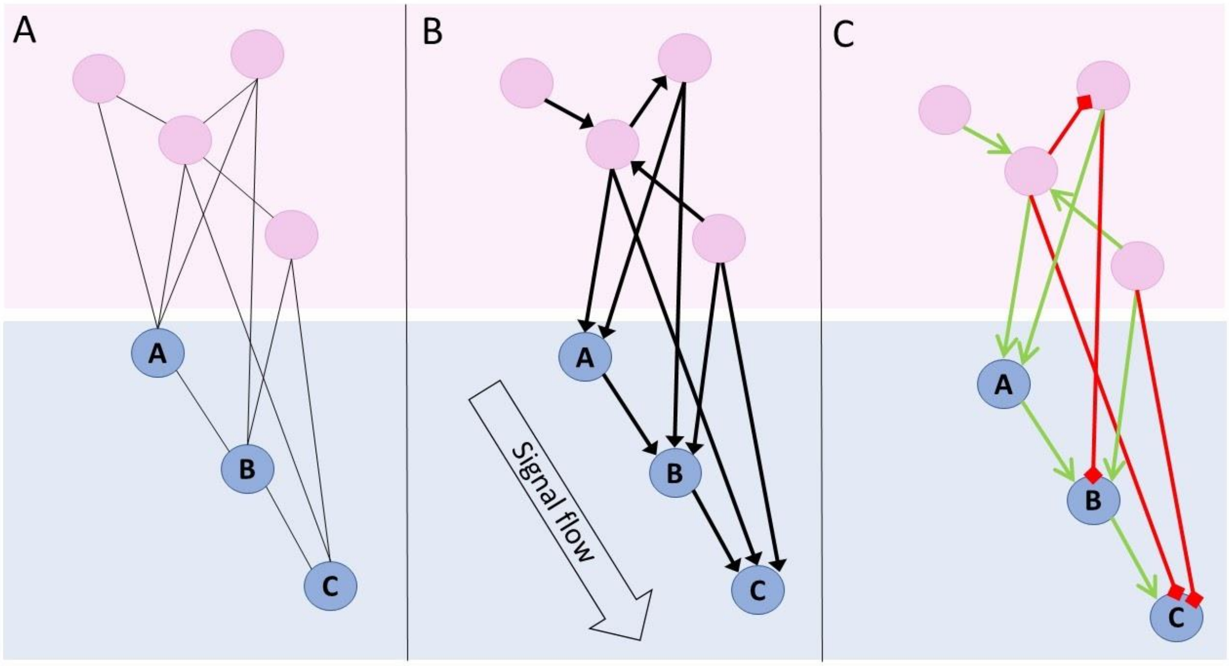

Supplementary Figure 1 - Direction and signage in signaling networks. The diagram illustrates the significance of knowing the direction and signage of interactions to provide a biologically relevant representation. With our direction and signage prediction, SignaLink3 provides a more detailed view of the regulatory mechanisms of signaling pathways. The upper section (pink) shows the PPI regulators of pathway member proteins A, B and C (bottom section, blue). **A, Undirected and unsigned network.** Interactions have no known direction or signage annotation. This representation does not carry information on which protein has an effect on which. **B, Directed but unsigned network.** By applying predicted direction scores to interactions integrated in SignaLink3, we supply the direction of previously undirected interactions which help in understanding the direction of signal flow. **C, Directed, signed network.** By applying predicted signage scores to directed interactions integrated in SignaLink3, we supply information of the effect one protein has on the other. Green arrows represent stimulatory, while red ones represent inhibitory interactions. With this knowledge the regulatory mechanisms can be traced, providing a more accurate and biologically relevant network representation.

Supplementary Table 1 - Comparison of database features. * - Provides a large option of files available for download of different subsets of the resource (*e.g.,* individual files for certain pathways or species), but limited customizability for interactions. Empty cells indicate that the feature is missing from the resource. NA - No available reported number of pathways

	**Reactome**	**ACSN**	**ConsensusPathDB**	**Pathway Commons**	**OmniPath**	**SIGNOR**	**SignaLink 2**	**SignaLink 3**
**Organisms**	16	only *H. sapiens*	3	only *H. sapiens*	only H. sapiens	3	3	4
**Contains manual curation**	Yes	Yes				Yes	Yes	Yes
**Contains integrated data**			Yes	Yes	Yes		Yes	Yes
**# of pathways (human)**	2536	13 'maps'	4593 'pathway gene sets'	4794	NA	NA	7	13
**Cross-talks and multi-pathway proteins**	Yes						Yes	Yes
**Directed PPIs**				Yes	Yes	Yes	Yes	Yes
**Post-translational modifiers**				Yes ('controls phosphorylation ')	Yes	Yes	Yes	Yes
**Transcriptional regulation**	Yes - in FI network	Few annotated	Yes	Yes ('controls expression')	Yes	Yes	Yes	Yes
**miRNA regulation**				Not available	Yes	Few	Yes	Yes
**lncRNA regulation**					Yes			Yes
**Tissue expression/subcellular localization**		Some cell type specific maps	GO cellular compartment					Yes
**Interactive network view**	Yes	Yes		Yes		Yes	Yes	Yes
**Customizable download options**	*			*	Yes, only through web service	*	Yes	Yes
**Search whole database**	Yes		Yes	Yes		Yes	Yes	Yes
